# The Ketamine Antidepressant Story: New Insights

**DOI:** 10.3390/molecules25235777

**Published:** 2020-12-07

**Authors:** Tahani K. Alshammari

**Affiliations:** Department of Pharmacology and Toxicology, College of Pharmacy, King Saud University, P.O. Box 2475, Riyadh 11451, Saudi Arabia; talshammary@ksu.edu.sa

**Keywords:** ketamine 1, antidepressant 2, rapid antidepressant 3

## Abstract

Ketamine is a versatile agent primarily utilized as a dissociative anesthetic, which acts by blocking the excitatory receptor *N*-methyl-d-aspartate receptor (NMDA). It functions to inhibit the current of both Na^+^ and K^+^ voltage-gated channels, thus preventing serotonin and dopamine reuptake. Studies have indicated that administering a single subanesthetic dose of ketamine relieves depression rapidly and that the effect is sustained. For decades antidepressant agents were based on the monoamine theory. Although ketamine may not be the golden antidepressant, it has opened new avenues toward mechanisms involved in the pathology of treatment-resistant depression and achieving rapid antidepressant effects. Thus, preclinical studies focusing on deciphering the molecular mechanisms involved in the antidepressant action of ketamine will assist in the development of a new antidepressant. This review was conducted to elucidate the emerging pathways that can explain the complex dose-dependent mechanisms achieved by administering ketamine to treat major depressive disorders. Special attention was paid to reviewing the literature on hydroxynorketamines, which are ketamine metabolites that have recently attracted attention in the context of depression.

## 1. Introduction

The history of ketamine began with “phencyclidine”, which was initially produced in 1956 by chemists working for the “Parke Davis Company” [1]. Ketamine, a short-term analog of the phencyclidine identified as CI-581, was produced at Parke Davis in 1962 by Calvin Stevens [2]. With a similar potential to anesthetics and less causative of delirium than its parent drug, phencyclidine, ketamine was selected for human trials in 1964 [3]. In 1970, Ketalar (owned by Par Sterile Products) emerged as the first sample of ketamine approved by the “Food and drug administration (FDA)” for use by humans [4]. The extensive therapeutic applications of ketamine make it one of the safest available anesthetic agents. Efficient analgesic and sedative effects can be reached at subanesthetic doses [5]. However, ketamine usage is associated with several dose-dependent drawbacks such as dizziness, nausea, vomiting, and hypersalivation [6]. It can also lead to psychological dissociation, which has limited its extensive use in clinical practice. Ketamine-induced psychological dissociation is characterized by altered feelings and perceptions, including auditory and visual delusions [7]. In addition, higher doses of ketamine may trigger schizophrenia-associated symptoms, including changed perceptions [8,9]. In acute usage of ketamine, these effects are resolved within 2 h; however, long-term use could cause several persistent and pronounced neuropsychiatric symptoms, including symptoms associated with schizophrenia, cognitive impairments, and a reduced psychological comfort zone [3,10]. Infrequent effects include cardiopulmonary toxic events, tachycardia, and hypertension [11,12]. Another serious effect is abuse liability. In rats, ketamine reaches anesthetic effects at a dose of 87 mg ketamine/kg [13]. The repeated administration of a subanesthetic dose (20 mg/kg for 2 months) reduced anxiety and depression-like behaviors in rats. This dose and duration did not cause dependence, which was measured by drug-seeking while performing place preference conditioning tasks [14]. Most of the ketamine dependence is achieved in clinical settings with doses higher than the ones used therapeutically [15]. Most of the illicit drug users utilize multiple drugs; however, a previous report demonstrated that, in a cohort of ketamine abusers only, subjects did not exhibit changes in their cognitive capacity. Of note, magnetic resonance imaging revealed changes in (11C)NNC112, an indicator of the dopamine D1 receptor, in multiple brain regions. The analysis suggested that the dopaminergic D1 receptor exhibits region-specific upregulation upon the recreational use of ketamine [16]. In vitro, a study has demonstrated that the activity of NMDA receptors is associated with the dopaminergic D1 receptor translocation and activation. This functional association has been shown to lead to an imbalance between the dopaminergic D1 and D2 receptor, which could provide the basis for psychiatric disorders, such as schizophrenia [17]. A previous study indicated that long-term ketamine use results in a noticeable reduction in the volume of the frontal gray matter. The magnetic resonance images indicated that this reduction was correlated with the dose and duration of ketamine use. However, most of these studies were conducted on polydrug users [16]. Cognitive assessment of aging studies revealed that the volume of the frontal gray matter was found to be essential in modulating cognitive capacity [18].

## 2. Ketamine as an Antidepressant

Although it was initially developed as an anesthetic, ketamine has exhibited other significant effects. For instance, during the last decade, it was recognized as an antidepressant. Despite its proven therapeutic efficacy, the underlying mechanisms are far from understood. Consequently, it has recently been the focus of considerable research attention. For instance, one study found that single-dose administration is associated with immediate antidepressant effects [14]. Clinical studies have also shown that the low-dose administration of ketamine reduces the functional connectivity of the subgenual anterior cingulate cortex [15], a brain region that postmortem studies have indicated is significantly reduced in depressed patients [16]. However, the functional effects are quite complex. One report found that the antidepressant effects of ketamine are associated with different types of alteration in brain oscillation. Specifically, theta and gamma oscillations were increased anteriorly, while theta, delta, and alpha oscillations were reduced posteriorly [17]. These oscillations are functionally distinctive. For example, the theta wave is associated with learning, the alpha wave is linked to sleep cycles, the beta wave is associated with emotional and cognitive tasks, and the gamma wave is linked to the sensory and cognitive capacity [19]. It is therefore important to determine the consequences of administering a subanesthetic dose of ketamine.

In addition, ketamine has been found to modulate glucose metabolisms in different brain regions associated with anxiety and depression [19], and is demonstrably effective in managing treatment-resistant depression [20]. Under physiological conditions, multiple mechanisms maintain energy supply to the neuronal populations. These dynamic mechanisms are affected by sleep, awakeness, and diseased states. Numerous psychiatric disorders have been reported to alter neurometabolic coupling, including depression. Based on postmortem tissue from depressed patients, proteomic study has reported >90 proteins involved in mechanisms regulating neurometabolic coupling. Implicating these homeostatic mechanisms in the pathology of depression [21]. Furthermore, functional imaging studies have supported the involvement of brain energy metabolism in the pathology of depression. Clinical settings have found that creatine phosphate and adenosine triphosphate levels were altered in multiple brain regions of depressed patients. In addition, the uptake of 18 (F)fluorodeoxyglucose, an indicator for glucose metabolism in neuronal populations, was found to be altered in multiple brain regions of depressed patients; these changes were reversed upon the administration of standard antidepressants. Of note, ketamine was found to reduce suicidal tendencies [14].

In the acute administration of ketamine, both synaptic connectivity and plasticity are potentiated. Preclinical studies have found that ketamine remedied the impairment in fear extinction memory in a model of depression [22]. In support of this finding, d-cycloserine, another NMDA partial agonist, was shown to modulate the extinction of fear [23]. The genetic mutations of GluN2B/NR2B, an NMDA receptor subunit, have been linked to brain disorders. For instance, hippocampal long-term depression (LTD) and the GluN2B protein level were substantially reduced in Grin2b-mutant mice. Behaviorally, these mutant mice exhibit anxiety-like phenotypes. The administration of d-cycloserine at an early age reversed these changes. This suggested that early interventions could be an excellent solution in terms of targeting NMDA-related depressive mechanisms [24].

It also indicated that, pharmacologically, ketamine has managed to reach molecular mechanisms that have not been reached by standard antidepressants. However, there are some limitations of ketamine as golden antidepressant. First, the existing clinical studies were conducted on small sample sizes with short follow-up periods for ketamine and other glutamate receptor modulators used to treat depression in adults [25]. Secondly, the cellular mechanisms are poorly understood. Thirdly, a recent study reported that long-term treatment with ketamine might alter brain topology [26]. This review was thus undertaken to examine recent studies that investigated potential mechanisms mediating the antidepressant effects of ketamine. The aim of this study was to summarize and highlight the fundamental mechanisms involved in the complex mechanisms underlying neuropsychiatric disorders.

## 3. Emerging Antidepressant Mechanisms

### 3.1. NMDA Receptors as Mediators

NMDA receptors are essential elements that modulate the fundamental mechanisms underlying learning and memory, including synaptic plasticity, long-term potentiation (LTP), and LTD [27]. Ketamine is a non-competitive NMDA receptor antagonist. The activation of NMDAR requires the simultaneous binding of “glycine, d-serine, and l-glutamate” at the corresponding subunits of GluN2 and GluN1, along with a voltage reliant magnesium (Mg^2+^) block repulsion at the pore of the ion channel through depolarization of the membrane, followed by to calcium influx [28]. Electrophysiological studies have demonstrated that a micromolar ketamine dose prevented NMDA synaptic events had a minimal impact on AMPA receptor excitatory events, and caused a persistent increase in principal cell spikes firing in the acute hippocampal slices of the population; it also mediated LTD [29].

Accumulating evidence indicates that the principal effect of a ketamine-mediated antidepressant is caused by the blocking of the NMDA receptor [30]. Using the forced-swim test, Trullas and Skolnick were first to recognize the low dose of ketamine-mediated antidepressant effects, as measured by immobility time [31]. This was achieved with a subanesthetic dose of 2–50 μm. These subsequently blocked the tonic glutamatergic input to the GABAergic inhibitory interneurons, resulting in glutamatergic disinhibition [32]. This led to an overall reduction in GABAergic-mediated feedback inhibition, which then resulted in elevated excitatory transmission-glutamatergic signaling [33]. Elevated glutamatergic signaling potentiates the ionotropic AMPARs, leading to an influx of sodium and the depolarization of the membrane potential [34]. The excitation–inhibition balance regulates neuronal excitability, communication dynamics, synaptic plasticity, and circuits wiring. A minute input delay would lead to a millisecond change in postsynaptic firing capabilities and an overall change in the amplitude and timing of the membrane depolarization in the hippocampus [35]. This hypothesis is an emerging risk for psychiatric disorders [36,37,38].

Electrophysiological studies have reported that the disinhibition hypothesis is a potential mechanism mediating the rapid antidepressant effect. Another study found that a low ketamine dose disrupted the excitatory–inhibitory balance in hippocampal pyramidal cells, primarily reducing synaptic mediated inhibition in the hippocampal area of acute brain slices. This increased synaptic-driven action potential without affecting the intrinsic excitability. In addition, functional changes of the disinhibition mechanism were observed while applying scopolamine, a muscarinic receptor antagonist, and GLYX-13, a partial NMDAR competitive agonist. Both agents were found to elicit antidepressant effects in preclinical settings [39]. This indicated the functional involvement of the disinhibition hypothesis in modulating rapid-acting antidepressant effects, and possibly treatment-resistant depression.

**Figure 1:**
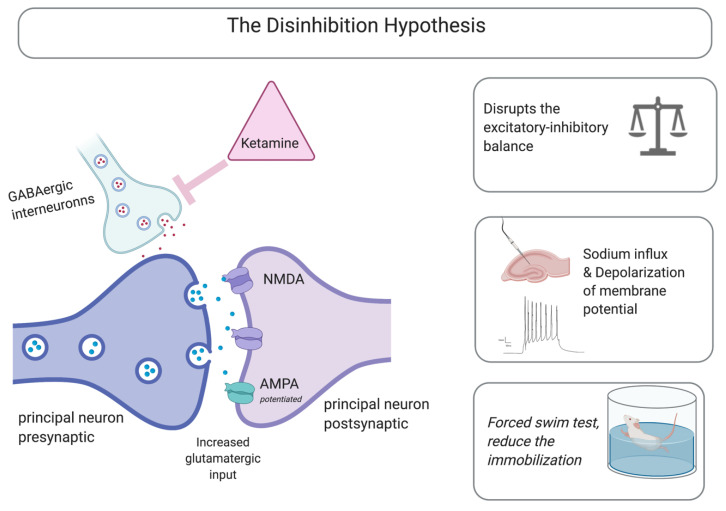
Main mechanisms described in the disinhibition hypothesis. Created with BioRender.

In another preclinical study, transgenic mice with selective mutations in GluN2B within GABAergic interneurons, ketamine failed to mediate its antidepressant effects [35]. Overall, based on the disinhibition hypothesis, it has been suggested that ketamine selectively blocks the NMDARs articulated on the GABAergic inhibitory interneurons [40]. However, other studies have contradicted this. For example, in mice with mutations of the GABAA receptor, a low ketamine dose had an antidepressant effect [41]. This indicated that the rapid antidepressant effect of ketamine is mediated by complex interconnected mechanisms, and that the disinhibition theory plays an important role in this process. Computational and pharmacogenomic network analysis of the rapid antidepressant effects of ketamine revealed the existence of single nucleotide polymorphisms (SNPs) associated with multiple genes. The analysis identified subnetworks that include the glutamatergic system, neuroplasticity, addition, and depression [42]. Figure 1 summarizes the main mechanisms described in the disinhibition hypothesis.

### 3.2. Non-NMDA Mediators

#### Neurochemical Cascades and Other Mechanisms

Other suggested mechanisms involve neurochemical cascades, such as the involvement of the glycogen synthase kinase-3 (GSK3) in mediating rapid antidepressant effects. A preclinical study indicated that a low ketamine dose blocks GSK3 in mice, and that this pharmacological event is indispensable in mediating rapid antidepressants. These findings have been explored mechanistically using GSK3 knock-in mice [43]. GSK3 is ubiquitously involved in electrical signaling, as it modulates different isoforms of the sodium channel [44,45,46]. Lithium, an FDA approved in treating mania and depression [47], was found to modulate GSK3. A previous report indicated that treating mice with lithium mimics led to molecular changes in GSK3^+/−^ heterozygous mice [48]. In line with this evidence, another report suggested that GSK3 and phosphorylated GSK3 were altered in the brain homogenate of mice exposed to chronic mild stress [49,50].

The mammalian target of rapamycin (mTOR) is another neurochemical signaling mechanism reported to mediate the rapid antidepressant effects of ketamine. In another preclinical report, a low ketamine dose was shown to quickly activate mTOR signaling. Conversely, blocking mTOR resulted in the suppression of ketamine associated antidepressant events at cellular and behavioral levels [51]. Genetic manipulations of mTOR using the adeno-associated viral vector were found to mediate depressive-like behaviors comparable to those reported in chronic and unpredictable mild stress animal models [52]. In liposaccharide-treated mice expressing all phenotypic features of depression, mTOR/p70S6K signaling was elevated in the brain homogenate. Similar findings were observed in in vitro studies using cortical and stem cell neurons [53]. These findings implicate this pathway in the complex pathological mechanism of depression.

Ketamine is also known to modulate TrkB signaling, where it activates the brain-derived neurotrophic factor (BDNF) via complex cascades involving α-amino-3-hydroxy-5-methyl-4-isoxazole propionic acid receptors (AMPARs). This activation has been documented in an abundance of preclinical studies exploring multiple brain regions [54,55,56,57], peripheral tissues [58], and in vitro systems [59]. Through such signaling, ketamine was found to accelerate the differentiation of newly born neurons in the dentate gyrus [60]. The pharmacological activation of the flox system in hM3Dq transgenic mice, a selective targeted newly born neuron excitability deactivation is achieved within the hippocampal circuitry. Therefore, newly integrated hippocampal neurons were the only ones that could not fire properly. This mechanistic study indicated that reduced newly born neuron excitability prevented antidepressant effects mediated by fluoxetine, indicating that adult hippocampal neurogenesis is required for mediating antidepressant effects [25]. Adult neurogenesis is a crucial process involved in neuronal plasticity [61], depression and reward circuitry [62], and memory [63], indicating that this selective pathway activation is a common feature in depression pathology.

An emerging hypothesis proposes that the rapid antidepressant effect of ketamine is explicitly linked to the suppression of NMDAR-mediated burst firing in the lateral habenula. This is a brain region that regulates reward-related processes and is linked to the negative signals in reward circuitry. Electrophysiological recordings have indicated that a small amount of electrical stimulation results in the potent inhibition of signaling [64]. The dopamine system is significantly involved in modulating the phenotypic signature of depression. For instance, it is functionally pivotal in mediating anhedonia [65]. Physiologically, neuronal populations in the lateral habenula exhibit remarkable burst firing and synchronized oscillation at the theta, and both features are associated with depressive-like behavior in rats. The administration of ketamine reverses these behavioral and electrophysiological phenotypes. Another study indicated that both NMDA receptors and low-voltage-sensitive T-type calcium channels (T-VSCCs) were involved in these actions [66]. In addition, a recent report examining the electrophysiological properties of ketamine on principal cell voltage-gated Na^+^ channels in the thalamocortical brain region suggested that ketamine sped up the inactivation and reduced the inactivation time following the recovery of Na^+^ channels [67]. Clinically, slow inactivation is an underlying feature of the basic mechanism of anesthesia [68]. Of note, functional imaging in clinical settings showed that the thalamic brain regions were linked to treatment-resistant depression [69].

Another suggested mechanism is the altering of Gαs subcellular localization by ketamine. For instance, in vitro studies have indicated that ketamine altered Gαs distribution, resulting in an overall increase in the available plasma membrane subunits and potentiation of the adenylyl cyclase coupling, which enhances intracellular cyclic adenosine monophosphate (cAMP). Therefore, the phosphorylation of the cAMP element-binding protein (CREB) is activated, elevating the expression of BDNF [70]. Furthermore, the administration of TAK-137, an AMPA receptor potentiator, is associated with the activation of neurochemical cascades, including the Akt, mTOR, p70 S6 kinase 1, and BDNF cascades. A 3-day administration of TAK-137 was pharmacologically sufficient to function as an antidepressant during novelty-suppressed feeding [71]. The enhanced S6K1 expression in the medial prefrontal cortex was also found to prevent depression-like effects in a rat model of depression [72].

In a chronic and unpredictable mild stress model, targeting the endocannabinoid system via the 2-arachidonoylglycerol pathway was found to alter the phenotypic signature of depression-like effects. Specifically, the administration of a monoacylglycerol lipase inhibitor activated the 2-arachidonoylglycerol pathway and, as a consequence, reversed changes in the depolarization of hippocampal CA1 principal cells, and behavioral changes [52].

Opioid (mu, delta, and kappa) receptors are also potential targets ketamine mediated rapid-antidepressant mechanisms [73]. For instance, ketamine attenuated suicidal tendencies through the modulation of opioid receptors. One study found that the administration of naltrexone, a non-selective opioid receptor antagonist, precludes ketamine-induced antidepressant effects [74]. In line with this finding, another study examined motivational behavior through the conditioned place preference of morphine, where the level of anxiety in mice was evaluated while they were performing open arm and sucrose preference tasks. The results revealed that ketamine reduced the anxiety-like effects and prevented morphine-induced conditioned place preference [75]. In line with this, buprenorphine, although it is limited its abuse liability, has shown better tolerability and efficacy profile as adjunctive therapy, especially in treatment-resistant depression [76].

A prenatal study indicated that exposure to an anesthetic dose of ketamine altered oxidative stress and autophagy [77]. Autophagy has attracted increasing attention in relation to brain disorders; it is a housekeeping process that maintains cellular homeostasis and can be activated by multiple factors, including exposure to stress [78]. In vitro studies also identified changes in the autophagy pathway following exposure to ketamine [77,79,80]. This indicates that ketamine anesthesia triggered autophagy in the hippocampus of the fetus and in PC12 cells [77]. A standard antidepressant, fluoxetine, was found to promote autophagosomes in mice exposed to chronic mild stress [81]. It is possible that the antidepressant actions of ketamine will reach and regulate the autophagy machinery; however, whether a subanesthetic ketamine dose impacts autophagy is yet to be determined.

In addition, ketamine modulates different components of the immune system [82]. At a high concentration, ketamine prevents endotoxin-induced pulmonary injury in rats exposed to lipopolysaccharides [83]. It has also been shown to modulate p38 mitogen-activated protein kinase (MAPK) in neutrophils [84]. Furthermore, acute treatment with MAPK kinase inhibitor, PD184161, has been found to lead to depressive-like behavior in rats and preclude ketamine-induced antidepressant-like effects while the rats perform forced swimming test tasks. At a molecular level, PD184161 reduced the phosphorylation of different MAPK kinase pathway components in brain regions associated with depression and anxiety, including the amygdala and nucleus accumbens [85]. In fact, different antidepressants were found to modulate the MAPK pathway in clinical and preclinical studies [86].

Reports have implicated the serotoninergic system in the rapid antidepressant mechanisms mediated by ketamine, although the exact mechanism is far from understood. One report indicated that ketamine failed to reduce the immobility time of transgenic mice lacking a serotonin transporter (SERT). Similar findings were observed in mice lacking the membrane monoamine transporter (PMAT). Of note, high-speed chronoamperometry performed during in vivo studies of the hippocampal CA3 indicated that ketamine prevented the clearance of serotonin in wild-type mice, but failed to achieve this in SERT or PMAT transgenic mice [87].

Another component involved in the ketamine-mediated antidepressant effect is the eukaryotic elongation factor 2 kinase (eEF2K). Previous reports have suggested that ketamine blocks the spontaneous neurotransmission mediated by NMDAR, affecting eEF2K inhibition, and thus preventing the phosphorylation of the eEF2 substrate. Such impact consequently leads to an enhancement in BDNF translation [88]. Both ketamine and memantine exert their actions mainly via NMDAR coupling. Memantine was found to lack the modulation capacity ketamine has in eEF2K, suggesting that eEF2K is a key element in mediating the rapid antidepressant effects of ketamine [89].

Clinical studies indicated that abnormalities in glial cells are a unifying feature in major depressive disorder [90,91,92]. A different study demonstrated that glial cells were reduced in postmortem tissue from depressed patients in the prefrontal cortex [93], anterior cingulate cortex [94], and amygdala [95]. In line with these findings, treatment with antidepressant agents rescued glial cell function [96]. Physiologically, glial cells communicate with neuronal populations in a bidirectional manner. In neuron-glia signaling, neuronal synapses release neurotransmitters, leading to the activation of glial signaling, which further leads to an increase in the level of Ca^2+^ intracellularly. This communication has been shown to trigger glia-glia signaling in a similar manner, which leads to an overall modulation of electrical firing, neuronal transmission, and synaptic plasticity [41,97,98,99]. The administration of an anesthetic dose of ketamine to a mouse cortex selectively reduces calcium signaling in astrocytes [100]. Of note, a recent study demonstrated that a low ketamine dose mitigated stimulus-evoked astroglia calcium signaling, indicating that ketamine-induced changes in astroglia calcium signaling might be involved in non-NMDAR-mediated mechanisms [101].

**Figure 2:**
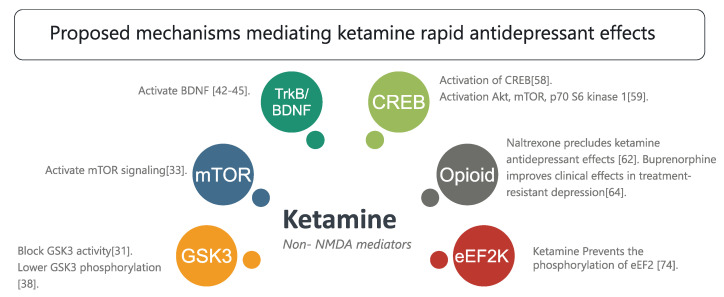
Main mechanisms underlying non-NMDA-mediated affects of ketamine.

In combination, these findings suggested that ketamine is ubiquitously involved in the modulation of different signaling pathways. The main mechanisms underlying non-NMDA-mediated affects of ketamine are summarized in Figure 2.

## 4. Metabolites

Ketamine undergoes extensive metabolism, primarily through cytochrome P450 isoforms. This process yields a number of metabolites, including hydroxynorketamines and (2*S*,6*S*;2*R*,6*R*)-hydroxynorketamines [68]. Biochemical analysis in clinical and preclinical studies has indicated that these are the primary metabolites of ketamine [69,70]. Although initial reports regarded hydroxynorketamines as inactive metabolites, due to their lack of the stimulating activities of hyperlocomotion activities during the recovery period of postanesthesia [71], later studies have shown that they are essential in mediating antidepressant effects [70]. Using multidisciplinary approaches, the (2*R*,6*R*)-hydroxynorketamines were found to mediate antidepressant effects [70] through mechanisms that are not NMDA-related. Previous reports have suggested that locomotion activities are sexually dimorphic following adolescence, and that this effect could be due to other ketamine and norketamine metabolites [72].

Another study reported that a ketamine-mediated antidepressant is sexually dimorphic, but this effect was not detected in ovariectomized female rats [73]. In addition, pharmacokinetic profiling revealed that, as compared with ketamine and its other metabolites (2*S*,6*S*;2*R*,6*R*), hydroxynorketamines were metabolized in a gender-dependent manner. In fact, the level was significantly higher in female mice, which may explain the sexual dimorphic mechanism of the antidepressant. Of note, the chemical alteration of ketamine through deuteration at C6, which does not alter its associative affinity for NMDAR, drastically reduced its in vivo metabolism to (2*S*,6*S*;2*R*,6*R*)-hydroxynorketamines. Such manipulation impeded the antidepressant actions of ketamine amongst mice, demonstrating that its metabolism to (2*S*,6*S*;2*R*,6*R*)-hydroxynorketamines is essential for both immediate and persistent ketamine antidepressant responses [74].

Hydroxynorketamine metabolites play a role in potentiating events mediated by α-amino-3-hydroxy-5-methyl-4-isoxazole-propionic acid (AMPA) receptors [75]. AMPAR are ionotropic transmembrane glutamatergic receptors, and the principal receptors mediating fast synaptic neurotransmission in the brain. They are also the pharmacological targets for multiple signaling pathways that regulate neuronal wiring, circuitry, and synaptic plasticity [76]. Functionally, they are required to mediate both LTP and LTD mutations of GluR1 and GluR2. AMPA receptor subunits are associated with abolishing both LTP and LTD [77]. Of note, AMPA was found to be an essential element in mediating the sustained ketamine-associated antidepressant effect. In preclinical settings, pretreatment with NBQX, an AMPAR antagonist, significantly reduced the immobility time during a forced swim test, suggesting that NBQX prevents the antidepressant-like effect of ketamine [78,81]. Exposure to corticosteroid, a stress hormone, changed the molecular trafficking of AMPAR, and that of the LTP [77]. Conversely, the administration of tianeptine, an antidepressant and a memory enhancement agent, prevented these corticosterone-mediated AMPAR redistributions [82]. However, one study indicated that the activation of AMPAR is not essential in mediating the (*S*)-norketamine antidepressant effects [83].

In other electrophysiological studies, (2*S*,6*S*;2*R*,6*R*)-hydroxynorketamines were found to competitively block the α7-nicotinic acetylcholine receptor and, as a consequence, suppress the acetylcholine-evoked currents at nanomolar concentrations. Ketamine itself, however, was incapable of these events. This indicated that hydroxynorketamines are α7-nicotinic acetylcholine receptor-negative allosteric modulators, which could partially explain their therapeutic effects [85].

Another study report indicated that the infusion of mTOR inhibitors (rapamycin and AZD8055) in the cerebral ventricles of mice exposed to chronic mild stress prevented the antidepressant effects mediated by *S*-ketamine but not by *R*-ketamine, indicating the involvement of complex mechanisms in the therapeutic effects mediated by ketamine enantiomers [102]. These findings suggested that the role of mTOR is not essential in mediating the antidepressant effect of (*R*)-ketamine. In line with these results, in neonatal dexamethasone-exposed mice, R-ketamine exhibited more potent antidepressant effects than those of S-ketamine [103].

## 5. Conclusions

In addition to serving as a form of anesthesia and modulating pain sensation, recent studies have demonstrated that ketamine has more specific functions, such as modulating specific receptors, anti-inflammatory actions, and inducing synaptic formation, all of which implicate ketamine in the management of a range of clinical conditions. Even though this suggests ketamine has excellent potential, this is limited by its abuse liability and dissociative properties. For that reason, preclinical studies have focused on deciphering the electrophysiological, behavioral, and molecular mechanisms involved in the antidepressant action of ketamine.

Both in vitro modeling and optical imaging fields were developed progressively. Future studies based on advanced tools and platforms, such as humanized three-dimensional models, organoids with autonomous developmental systems and the blood–brain barrier microvessel-on-a-chip would promote answering mechanistic questions [104,105].

An understanding of those mechanisms underlying the antidepressant actions of ketamine would not only provide vital insights into the neurobiology of major depression, they would also enhance recognition and awareness of new and innovative therapeutic targets for developing the next generation of fast-paced antidepressants, which may lack side effects and exert more rapid effects. The inhibition of NMDAR has severe drawbacks, even at lower and subanesthetic doses, that increase the requirement of long-term use of the agent; this, in turn, entirely blocks the receptors, making it impractical to use for the treatment of depression.
